# Parametric generation of three-dimensional gait for robot-assisted rehabilitation

**DOI:** 10.1242/bio.047332

**Published:** 2020-03-05

**Authors:** Di Shi, Wuxiang Zhang, Xilun Ding, Lei Sun

**Affiliations:** 1 School of Mechanical Engineering and Automation, Beihang University, Beijing 100191, China; 2 Beijing Advanced Innovation Center for Biomedical Engineering, Beihang University, Beijing 100191, China; 3 Beijing Institute Traumatology & Orthopedics, Beijing Jishuitan Hospital, Beijing 100035, China

**Keywords:** Gait analysis, Gait generation, Robot-assisted rehabilitation, Regression analysis, Three-dimensional gait

## Abstract

For robot-assisted rehabilitation and assessment of patients with motor dysfunction, the parametric generation of their normal gait as the input for the robot is essential to match with the features of the patient to a greater extent. In addition, the gait needs to be in three-dimensional space, which meets the physiological structure of the human better, rather than only on a sagittal plane. Thus, a method for the parametric generation of three-dimensional gait based on the influence of the motion parameters and structure parameters is presented. First, the three-dimensional gait kinematic of participants is collected, and trajectories of ankle joint angle and ankle center position are calculated. Second, for the trajectories, gait features are extracted including gait events indicating the physiological features of walking gait, in addition to extremes indicating the geometrical features of the trajectories. Third, regression models are derived after using leave-one-out cross-validation for model optimization. Finally, cubic splines are fitted between the predicted gait features to generate the trajectories for a full gait cycle. It is inferred that the generated curves match the measured curves well. The method presented herein gives an important reference for research into lower limb rehabilitation robots.

## Abstract (precis)

**Summary:** A method for the parametric generation of three-dimensional gait based on the influence of the motion parameters and structure parameters is presented.

## INTRODUCTION

During the last decade, various efforts have been devoted to the development of lower limb rehabilitation robots. The robots imitate the human gait to treat the abnormal and/or pathological gait with robot-assisted rehabilitation, and the patients learn the gait pattern imposed on them by the robot-assisted rehabilitation (Luu et al., 2011). A normal gait pattern is often needed as the reference for the robot-assisted rehabilitation and assessment of the gait. Firstly, if the actual gait measured during walking deviates from the reference trajectory, the robot will aid the limbs to drive the actual gait back based on the assist-as-need (ANN) control strategy. Secondly, the effect of the rehabilitation is assessed by comparing the actual trajectories measured by the robot or other devices during and after rehabilitation with desired trajectories.

It is difficult to achieve the normal ankle motion of the patients directly by a motion-capture system for lower limb impairment. Pre-record trajectories from unimpaired participants are often used to create a reference pattern for patients, which also appears to be the most suitable approach (Koopman et al., 2014). With the recorded kinematic data, the trajectory is generated by the average method among different participants (Al-Obaidi et al., 2003; Kwon et al., 2015). However, this method has certain considerations that limit its use to specific applications. First, specific patients with different motion parameters (MPs) and structure parameters (SPs) exhibit different gait features. The use is constrained to a limited number of participants, and is not well-matched with specific patients. Next, the sagittal gait is primarily analyzed and generated (Hanlon and Anderson, 2006; Koopman et al., 2014; Lelas et al., 2003; Palmer and Lars, 2002), regardless of the motion in the horizontal and transverse planes in which the abnormal gait often occurs (Levinger et al., 2010; Presedo et al., 2016; Rethlefsen et al., 2006; Whelton et al., 2017; Yamamoto et al., 1994), for robot-assisted rehabilitation after curve-fitting and parameter optimization (Koopman et al., 2014; Luu et al., 2014). There are several reasons for this. First, most of the motions occur in the sagittal plane, so the lower limb rehabilitation robots are mainly designed to achieve the sagittal motion (Banala et al., 2009; Duschau-Wicke et al., 2010). However, human locomotion is a three-dimensional motion accomplished by the coordination of the lower limb. It also occurs in the transverse and frontal planes besides the sagittal plane to achieve foot progression, balance and stability of the body. It is necessary for lower limb rehabilitation robots to mimic the motion of real humans. By considering the motion only in the sagittal plane as the gait that a patient is recovering, the patient cannot regain the motion in accordance with his or her normal gait. Currently, some new types of rehabilitation robots that move actively in a three-dimensional space have been developed (Zhang et al., 2016, 2018), thus, require the need for the generation of the three-dimensional human gait for controlling a lower limb rehabilitation robot. Thus, a parametric method to generate the three-dimensional ankle motion is the solution to satisfy the needs for a specific patient. First, the MPs and SPs reflecting the motion and structure features of the human are selected. Subsequently, some gait features dependent on the MPs and SPs are analyzed. Further, the trajectories are generated by piece-wise curve fitting between every consecutive gait feature, such that when the MPs and SPs are determined, a tailored ankle motion is generated for them.

The primary problem is the selection of the MPs and SPs for parametric generation. Walking speed is a crucial MP. As the speed increases, the range and peak values of the ankle joint angle increase (Chehab et al., 2017; Koopman et al., 2014; Lelas et al., 2003; van Hoeve et al., 2017). For the SP, previous studies primarily focused on the effect of body height on the motion of the ankle and did not find significant correlation between body height and any of their kinematic parameters when using a normalized walking speed (normalized to body height) (Lelas et al., 2003), by using a stepwise regression in the regression model that includes body height (Koopman et al., 2014), or by analyzing the body mass index (BMI) (Chehab et al., 2017). When analyzing the motion of the ankle, SPs are selected according to the effect on the hip joint and knee joint, such as body height, but the length and width of the foot are seldom considered. Additionally, in three-dimensional gait, the foot progression angle (FPA) is another important parameter. Two variables of the lower extremity that have been shown to contribute to the direction of the FPA in adults are femoral torsion and tibial torsion (Andrews et al., 2010; Lee et al., 2013; Seber et al., 2000), while foot posture also contributes (Buldt et al., 2015). Thus, the FPA can be treated as an SP reflecting the structure of the lower limb and foot. Previous studies focused on the toe-in and toe-out gaits changing the FPA, and were proposed to be compensatory mechanisms as various kinematic adaptations (Ho et al., 2000; Jenkyn et al., 2008; Khan et al., 2017; Presedo et al., 2016). However, even healthy individuals exhibit various FPAs (Seber et al., 2000). Currently, the FPA during gait can be predicted using clinical measures in healthy participants (Cibulka et al., 2016). Thus, FPA is a type of generalized SP and should also be considered. The selection of gait features is important. Some gait features of the sagittal plane parameters are dependent on speed (Chehab et al., 2017; Koopman et al., 2014; Lelas et al., 2003); therefore, the peak values were used to parameterize and reconstruct the joint trajectories and were selected as gait features. However, they were chosen based on the geometrical features of the curves, regardless of the functional tasks required for walking gait. The angle value during the gait event should also be considered because the duration of the sub-phase is also influenced by the MPs and SPs (Hebenstreit et al., 2015).

In addition to joint angles, ankle center position (ACP), the position of ankle joint center relative to pelvis, is also a kind of reference for the robot-assisted rehabilitation. In a study, the ACP in the sagittal plane was used as the reference trajectory (Zanotto et al., 2013), and the participants viewed a target foot-trajectory on the screen with which they attempted to match their own foot-trajectory (Ranganathan et al., 2016). If the lower limb can be treated as a manipulator, the ACP is the position of the end effector. The human gait can be analyzed by robotics, and the ACP can be treated as the motion in the coordination space and the motion of the hip and knee joint are the motion in the joint space. Taking the length of the leg besides joint angles into account, the ACP can be calculated. So, the effect of body height on human gait can be revealed by the analysis of the ACP. At the same time, through the study of ACP characteristics, the overall impact of MPs and SPs on gait can be revealed.

The objective of this study is to present and evaluate a novel method for the parametric generation of three-dimensional gait, including ACP and ankle joint angle (AJA), for robot-assisted rehabilitation. Walking speed was selected as MP. Body height, the FPA, foot length and foot width were selected as SPs. The relative timing, angle and angular velocity were predicted based on the MP and SPs using regression models. LOOCV (leave-one-out cross-validation) was used for the optimization of the regression models, upon which a method to generate the human gait was based and presented. The method presented herein will facilitate understanding the motion during the gait associated with the MPs and SPs and can be used to generate gait-for-gait rehabilitation and assessment.

## RESULTS

### Regression models

**Fig. 1.**
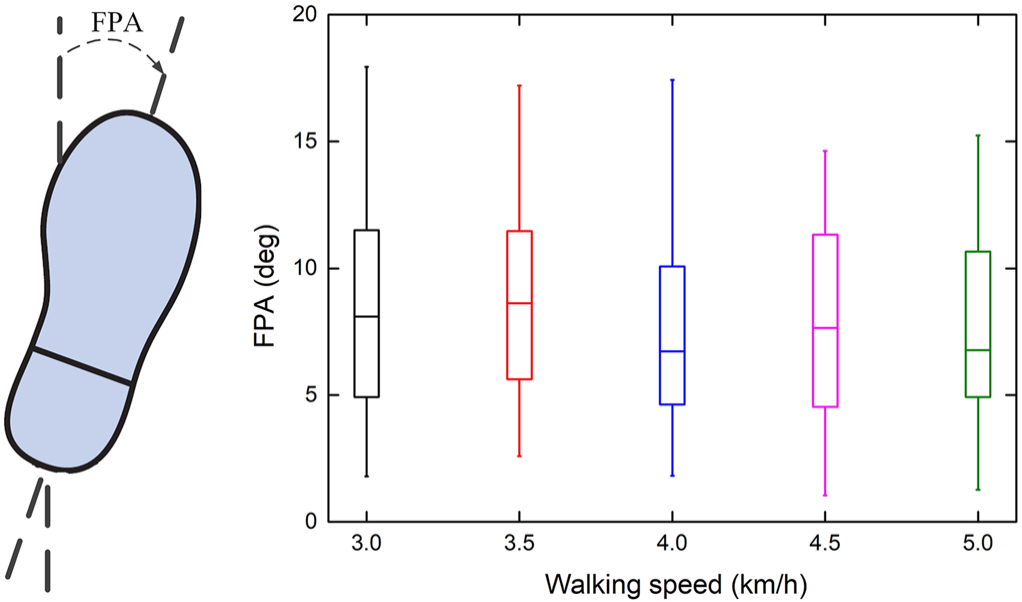
**Boxplots of individual mean durations in percent of the total gait cycle versus speed.** The curves represent the linear regression lines. The results of Wilcoxon matched pairs signed rank tests with Bonferroni correction are shown for each adjacent speed pair (*P<0.00076).

The FPA for all the participants at different speeds was positive (Seber et al., 2000). The ANOVA result of the FPA during different speeds demonstrated that the FPA did not change with walking speed (Fig. 1), indicating that FPA was only dependent on the structure of the lower limb and foot, regardless of MPs.

The regression models showed that the ACP was dependent on at least one gait factor (Tables 1 and 2). However, the peak values and relative timing of the velocity was not significantly affected by gait factors. In the non-parametric test, most gait features showed a dependency on walking speed, whereas body height and FPA affected the parameters of gait features to a lesser extent. Out of the 38 gait features, 26 were dependent on walking speed, whereas 15 and 8 gait features were dependent on body height and FPA, respectively. For instance, according to the regression model, the minimal position of the z axis (Zf1) was only dependent on body height and exhibited a decrease with the increase in body height owing to the increase in the range of motion on the position of the z axis. It was also illustrated that the duration of the phase and position of the y axis were affected by the FPA. According to the results of LOOCV, the regression equation expression of walking speed is a cubic relationship of the highest order, while other factors in the regression equation exhibit linear relations. Out of the 40 gait features, 23 were dependent on at least one parameter. Only four features were dependent on walking speed (cycle, DSP2, A1f2 and dA1e5) as shown in Tables 1 and 2, whereas 10 and 6 gait features were dependent on body height and FPA, respectively. Twelve gait features were dependent on foot length and foot width.

**Table 1.**
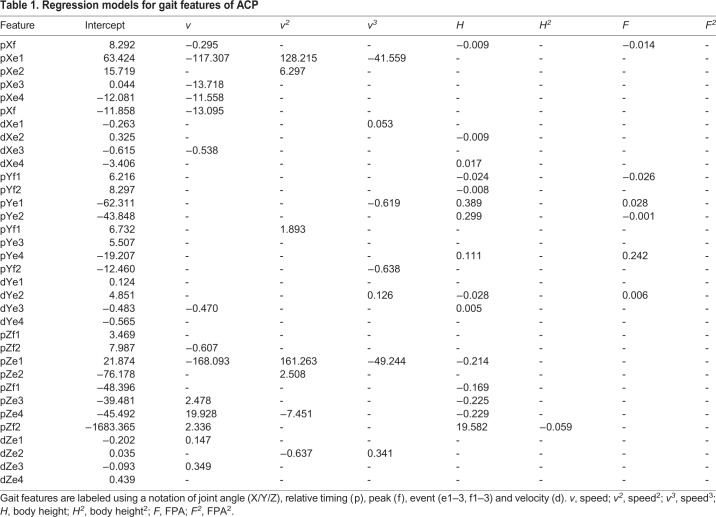
**Regression models for gait features of ACP**

**Table 2.**
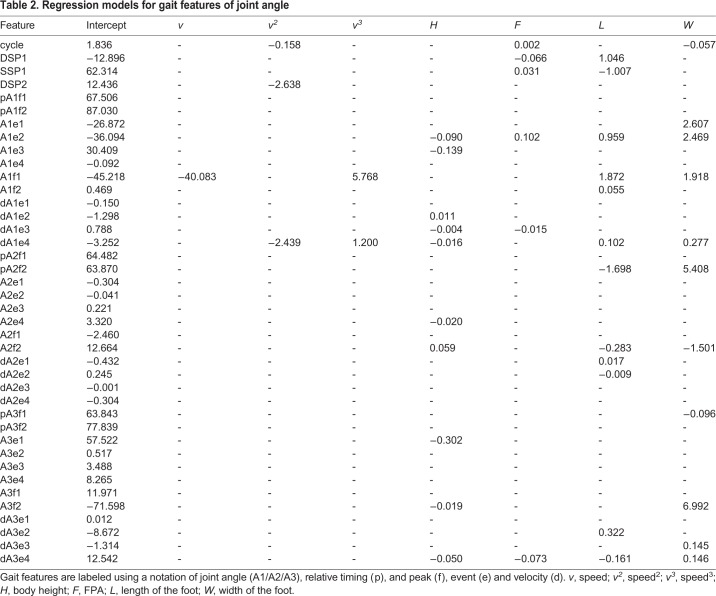
**Regression models for gait features of joint angle**

### Validation

**Fig. 2.**
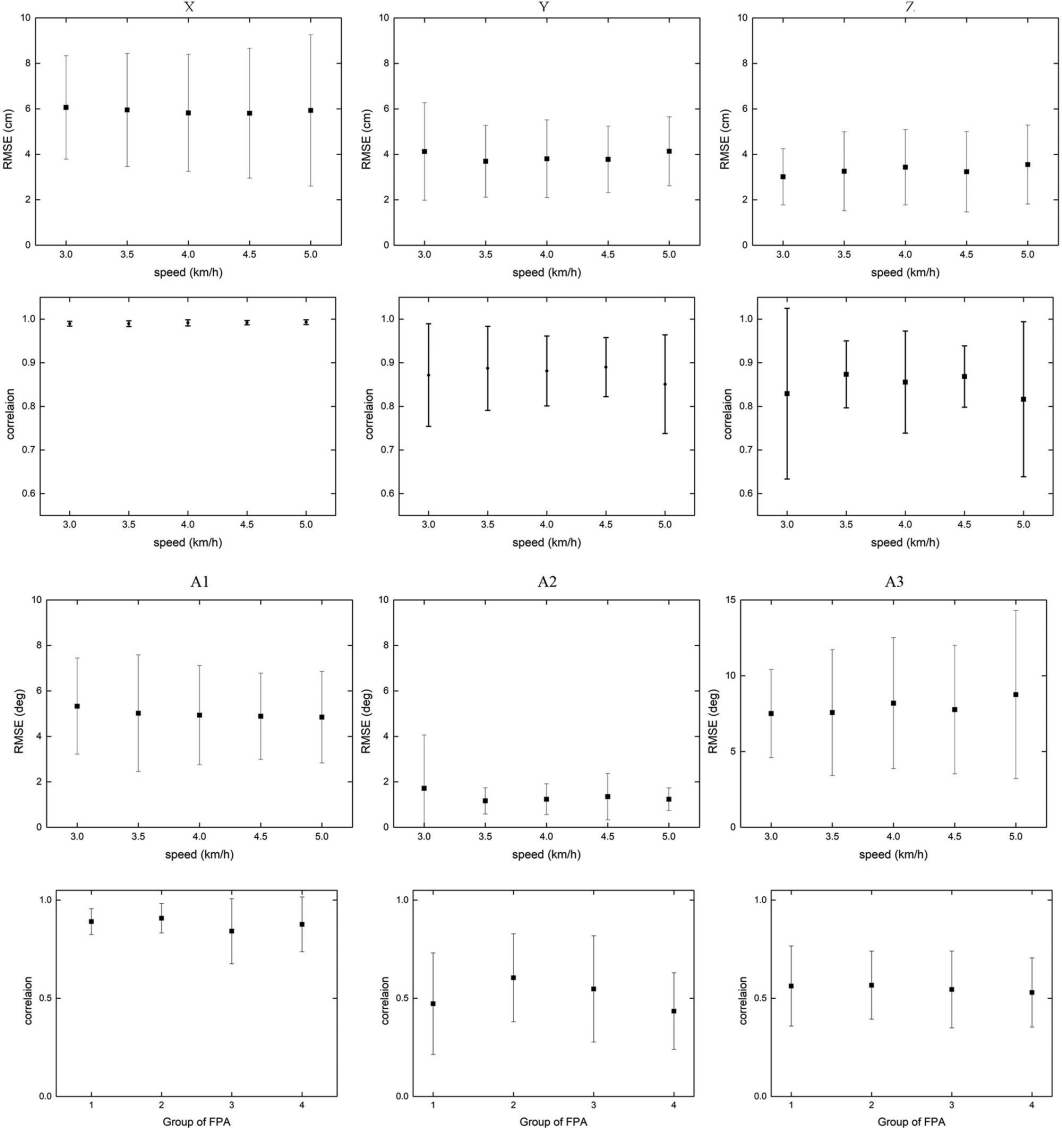
**Validation of the generated reference trajectories.** RMSE and correlation coefficients between actual and generated trajectories are averaged across participants for each walking speed.

With the obtained regression model, a set of reference trajectories was generated for each subject. The generated patterns matched the measured data well. Meanwhile it was found that the overall error does not change with walking speed, in a normal range. Root mean square error (RMSE) was used to reflect the quality of the fit shown in Fig. 2. For X, Y and Z, the RMSEs (averaged over all walking speeds) were 5.91 cm, 3.91 cm and 3.30 cm, respectively. Additionally, correlation coefficients were used to quantify the similarity between the actual and generated reference trajectories. For X, the correlation coefficient was above 0.97 for all walking speeds. For Y, it was above 0.51 for all walking speeds. For Z, it was above 0.51 for all walking speeds. The generated patterns matched the measured data well. RMSE and correlation coefficients were used to reflect the quality of the fit shown in Fig. 2. The spline fitting methodology was also compared to the traditional averaging method, where we calculated the average trajectories across the participants. Generally, the amplitude of the average trajectories was smaller than the amplitude of the generated trajectories. Further, the generated trajectories were compared with the trajectories, parameterized only by speed and body height (Figs S2 and S3).

## DISCUSSION

In this study, we have presented a novel method to parameterize and generate three-dimensional ankle motions. The method was based on fitting cubic splines between different gait features, which were estimated with regression models after optimization by LOOCV. From the analysis of the selected gait features, the regression models indicate that SPs have a larger effect than the MP on the selected gait features and the durations of the gait phase are significantly influenced by the SPs.

### Effect of parameters on gait features

One finding is that SPs have a larger effect than the MP on the selected gait features. In a previous study, body height had a limited effect on gait features compared with walking speed. It was revealed that walking speed and body height were related and as an increase in body height increased step length, walking speed increased adaptively. Thus, at the same walking speed, the subjects with different heights have different senses of fast and slow speeds, which was verified by the subjects participating in the experiment. The previous study focused on the joint angles and selected the hip and knee joint angles as the features (Koopman et al., 2014). The result revealed that walking speed had a significant effect on hip and knee joint angles. In this study, the ACP was analyzed relative to the pelvis to obtain a comprehensive understanding of the human gait. The ACP motion can be considered to be in coordination, whereas the motion of the hip and knee joint is in the joint space. The ACP can be calculated by considering the length of the leg besides the joint angles. Walking speed affected all gait features for the positions on the x and z axes. Increase in walking speed implies an increase in the amplitude of the positions on the x and z axes because of the increase in stride length and foot height (Stansfield et al., 2006). In our study, the effect of the structure parameters on gait was analyzed. According to the regression model results, gait features of the positions on the y and z axes were independent of body height, whereas amplitude increased with an increase in height. Moreover, the peak in the position on the z axis showed a significant dependence on walking speed due to the occurrence of Zf1 as a result of the knee exhibiting an extension peak and the leg being maintained straight.

**Fig. 3.**
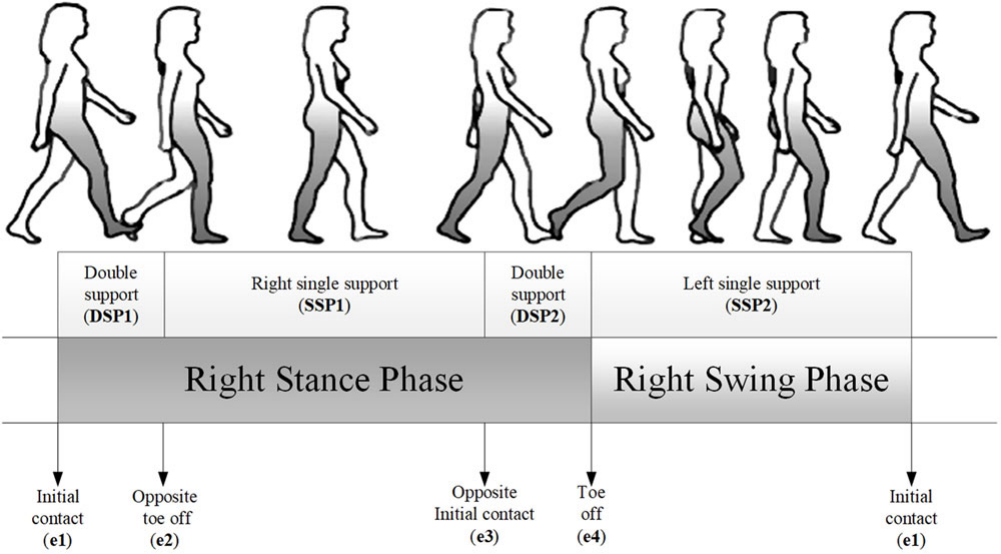
**Overview of the gait cycle and sub-phases analyzed in this study.** Total gait cycle, first double support phase (DSP1), first single support phase (SSP1), second double support phase (DSP2), initial-contact, opposite toe-off, opposite initial-contact, and toe-off.

**Fig. 4.**
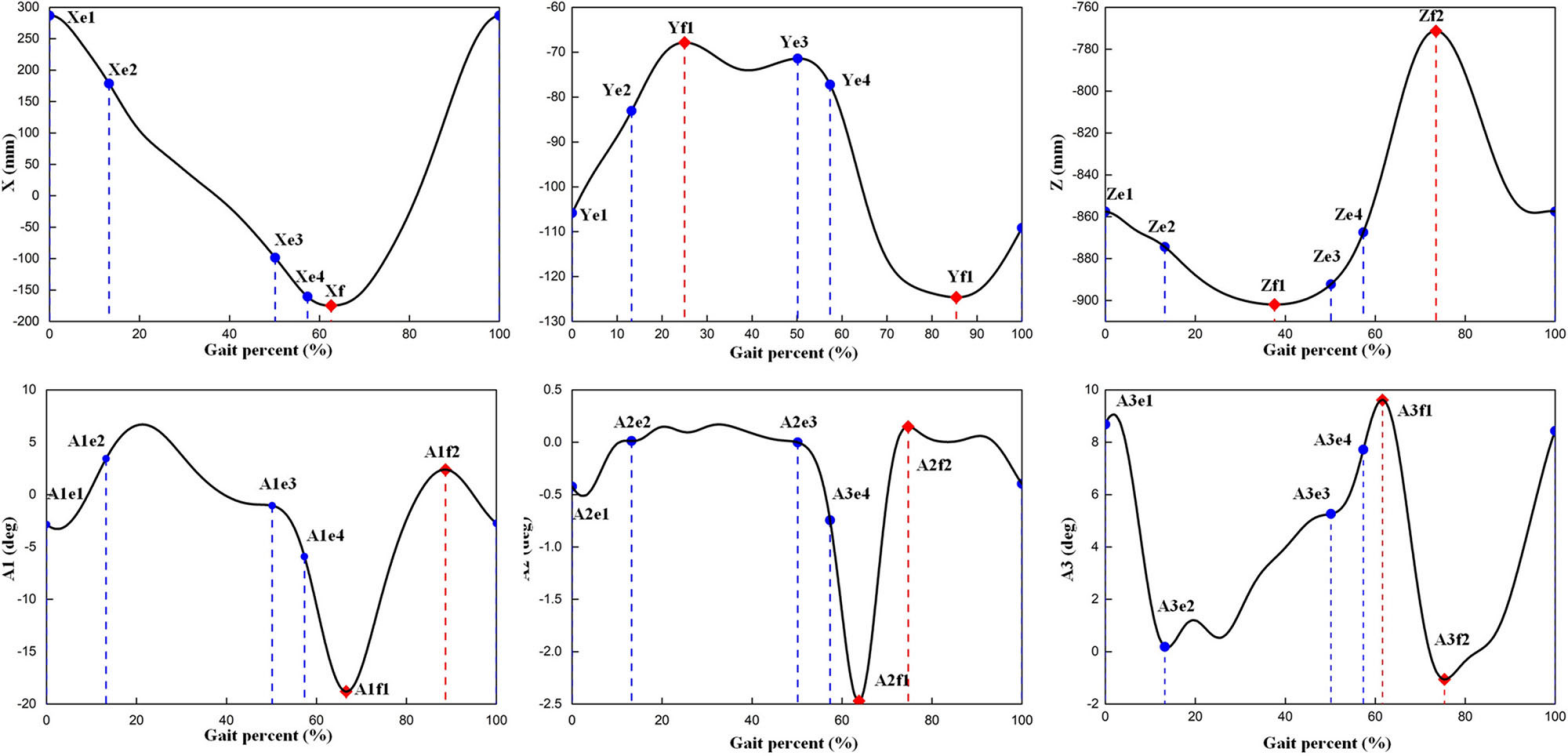
**Selection of gait features.** The dots indicate the amplitude of the gait event (e1, e2, e3 and e4) and the extreme values of the position (f1 and f2), all extracted as the gait features. A1, A2 and A3 indicate dorsiflexion (+)/plantarflexion, inversion (+)/eversion and internal (+)/external rotation angles of the ankle joint.

In this study, both the values when the peak and event occurred were selected as gait features (Figs 3 and 4). From the analysis of the selected gait features, the regression models were established and indicated that the SPs have a larger effect than the MP on the selected gait features. The previous study found that some of the sagittal plane gait features were primarily affected by walking speed (Hanlon and Anderson, 2006; Lelas et al., 2003; Stansfield et al., 2006). However, gait features in the transverse and horizontal planes, especially for the ankle joint, were seldom considered. Most of the studies indicated that body height presented a smaller effect on gait (Hanlon and Anderson, 2006; Lelas et al., 2003). However, in our study, it was illustrated that body height, along with foot length and foot width, influenced the dorsiflexion/plantarflexion angle (A1) primarily in the stance phase. This is because during the stance phase, the foot is in contact with the ground, whereas the lower limb and foot establish a kinematic chain. The ankle motion was only generated in the sagittal plane in the previous study (Feng et al., 2008). To establish the model of the lower limb, the thigh, shank and foot were treated as links, and they formed a kinematic chain during human gait. Therefore, the length of the foot is also an SP, similar to body height or the length of the thigh and shank. Moreover, for the three-dimensional gait, the foot is a rigid body; therefore, not only the length but also the width of the foot should be incorporated in the regression analysis as SPs. Walking speed indicated a smaller effect on the selected gait features, thereby indicating the importance of considering the SPs. In fact, the previous study did not consider the effect of walking speed when analyzing the foot kinematic (Buldt et al., 2015, 2013; Neal et al., 2014; Nester et al., 2007).

Gait cycle was affected by body height and FPA. Previous research revealed that gait cycle was impacted by walking speed (Hebenstreit et al., 2015). In our study, stride length decreased with an increase in body height and FPA, and both factors are related to the structure parameters. Additionally, FPA was affected by foot posture (Buldt et al., 2015) and foot loading pattern (Rosenbaum, 2013).

Another finding is that the durations of gait phases are significantly influenced by SPs. The previous study only analyzed the effect on relative duration regardless of the SPs. From the regression model, DSP1 is only influenced by SPs. DSP1 reflects the duration of the loading response. During this period, the foot is lowered to the ground by the plantarflexion of the ankle and at the end of the loading response, the foot is in complete contact with the ground; therefore, the smaller the FPA, the shorter the foot length, and the shorter the duration of the loading response. Meanwhile, the FPA primarily affected the DSP1 and SSP1, which was illustrated in a previous study, because the FPA is calculated through the stance phase (Simic et al., 2013). The stance phase can be divided into three phases: DSP1, SSP1 and DSP2. Our study indicates that the decrease was caused by the duration of DSP2.

### Comparison with traditional methods

The trajectories were generated based on regression models. Gait features that were influenced by at least one parameter were predicted by MPs and SPs. For gait features that were not influenced by any parameter, regression models were the average values among the participants. The results in our study indicate that the selected peak values of the three-dimensional trajectory – besides the peak in the sagittal plane (Hanlon and Anderson, 2006; Lelas et al., 2003; Stansfield et al., 2006) – during the swing phase could be predicted by MPs and SPs. For the selection of gait features, the peak indicating the geometrical features of the trajectories (Koopman et al., 2014) and gait event indicating the physiological features of walking gait were extracted. The number of gait features was reduced, because the relative timing of the gait events was the same for A1, A2 and A3. As previously mentioned, most studies that report normative gait patterns utilize individual normalized datasets. In our study, the AJA was parameterized by MPs and SPs (Fig. S4). Moreover, the generated trajectory of A1 was compared with the average trajectory in the previous study (Koopman et al., 2014); in our study, the trajectories of A2 and A3 were also compared. Further, the generated trajectories were compared with the trajectories parameterized only by speed and body height, and they demonstrated a more accurate effect.

As mentioned earlier, in most studies, the ACP was calculated by a two-link model in the sagittal plane using the joint angle in that plane. However, the joint angles were calculated by the orientation of the segment of the lower limb based on the Euler angle or fixed Euler angle (Kadaba et al., 1990). The foot position was calculated by the two-link model in the sagittal plane (Banala, 2007; Zanotto et al., 2014) using the joint angle in the sagittal plane. However, the joint angles were calculated by the orientation of the segment of the lower limb based on the Euler angle or fixed Euler angle (Kadaba et al., 1990). So, a method to calculate ACP directly rather than indirectly is necessary for research. The trajectory of the ACP can be obtained from the data collected by the motion capture system, but this trajectory is often relative to the coordinate of the world, {W}. The homogeneous coordinate transformation can be used to transform coordinates for the trajectory from the world to the segment of the human. Previously, the coordinate system of the human has been established according to the anatomy of human body (Banala et al., 2009). This way of establishing the coordinate system is usually only aimed at the sagittal plane. For three-dimensional trajectory, the coordinate system needs to be established based on the three-dimensional movement of human body. According to our study, ACP can be acquired based on MPs and SPs directly.

### Utility

Nowadays, the AAN control concept has become one of the prevailing paradigms to encourage patients' active participation during robot-assisted rehabilitation. The strategies are achieved by force-field control or impedance control based on position error, introducing a compliant virtual wall, which was developed to keep the patient's legs within a ‘tunnel’ around the desired gait trajectory. However, the force field is based on sagittal motion. Obtained regression models can be used to generate three-dimensional ACP as reference for trajectory. Using the ACP, a force-field control for three-dimensional gait adaptation using a lower limb rehabilitation robot can be established (Shi et al., 2019).

### Limitations

This study had some limitations. First, our results only explained the trajectory within a limited speed range (3, 3.5, 4, 4.5 and 5 km/h). Second, this study included only young healthy subjects without any lower limb injuries. Whether these gait trajectories could directly be used for elderly people is still unclear. For future studies, it would be beneficial to further investigate the relationship between structure parameters and gait features in detail.

### Conclusion

In this study, we presented a novel method to parameterize and generate three-dimensional gait based on analysis of walking gait. The method was based on fitting cubic splines between different gait features, which were estimated with regression models after optimization by LOOCV. The obtained regression models also indicated that SPs had a larger effect than walking speed on the selected gait features. From the RMSE and mean correlation coefficient between the generated and measured trajectories, it was inferred that the generated curves matched the measured curves well. The method presented herein will facilitate understanding the motion during gait associated with MPs and SPs and can be used to generate gait based on the MPs and SPs of the subject for robot-assisted rehabilitation and the assessment of human gait.

## MATERIALS AND METHODS

### Subjects and materials

25 healthy adults (19 men, 6 women, aged 23.76±2.81 years; height: 168.81±5.64 cm; BMI: 21.80±2.11 kg/m2) with no symptoms of orthopedic or neurological disorders volunteered for this study. The participants gave written informed consent, and study procedures were conducted in accordance with the Declaration of Helsinki.

The participants walked on a treadmill, starting with a familiarization period of 30 s followed by a 2-min walking trial. This was repeated at five different speeds (3, 3.5, 4, 4.5 and 5 km/h) according to the mean normal self-selected gait speed (Al-Obaidi et al., 2003) with 30 s breaks between trials. No specific instructions on how to walk on the treadmill were given. In accordance with the Helen Hayes marker set (Kadaba et al., 1989), 19 reflective markers with a diameter of 20 mm were attached to specified locations.

### Data analysis

#### Data processing

**Fig. 5.**
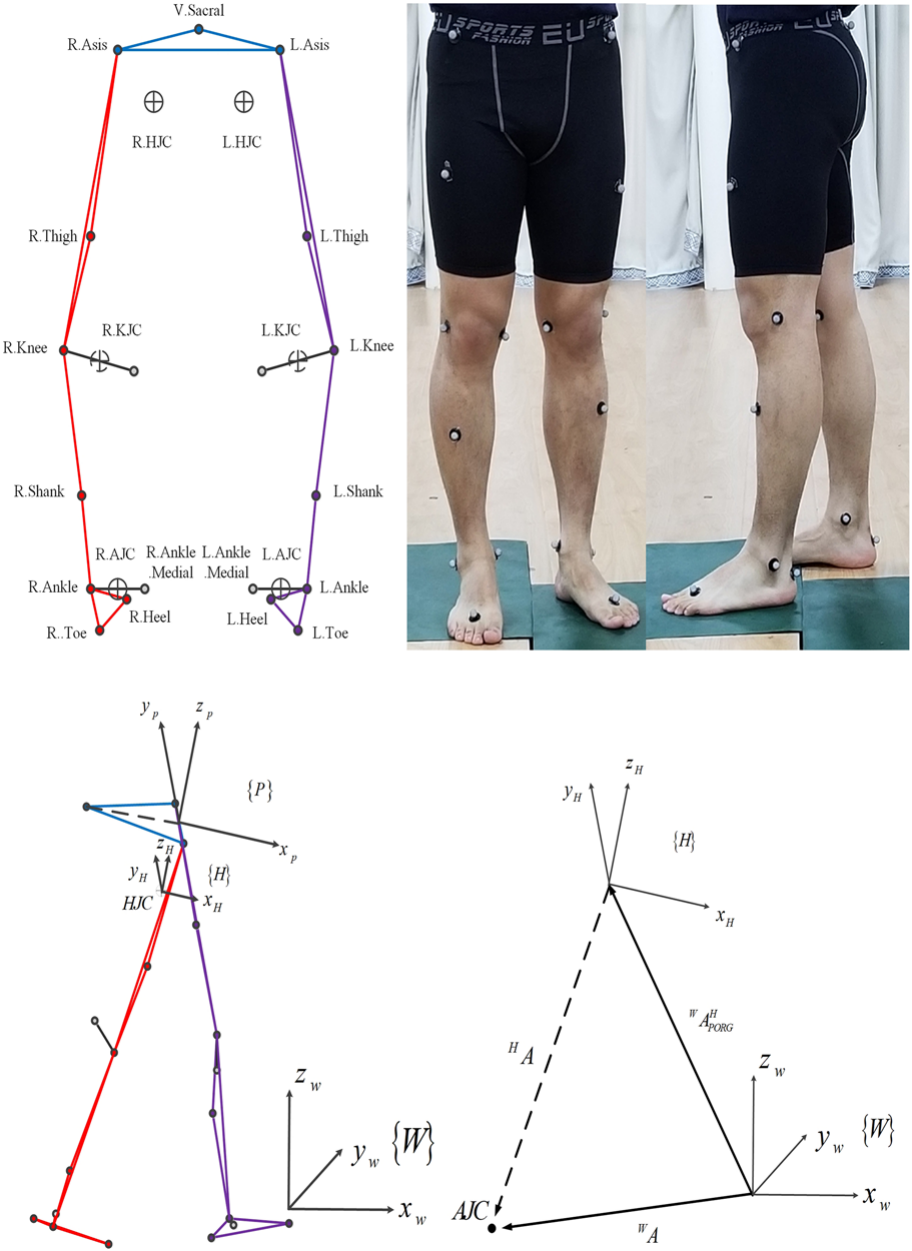
**Helen Hayes marker set.** To track the motion of the subject, 19 reflective markers with a diameter of 20 mm were affixed to specified locations. The hip joint center (HJC), knee joint center (KJC) and ankle joint center (AJC) of both legs were calculated using the Cortex software package.

Three-dimensional marker trajectories were collected with Cortex software (Motion Analysis Corporation, Santa Rosa, USA) using a motion analysis capture system with six digital cameras (Eagle cameras; Motion Analysis Corporation), collected at 100 Hz. The AJA and ankle joint center (AJC) of both legs were determined using Visual3D software (C-Motion, Inc., Germantown, MD). A homogeneous coordinate transformation was used to calculate the ACP. The hip joint center (HJC), knee joint center (KJC) and AJC of both the legs were calculated using the Cortex software packages. The ACP was the trajectory of the AJC in three-dimensional space and was directly obtained in the coordinates of the world (van Kammen et al. 2016), calibrated by a motion capture system. A homogeneous coordinate transformation was performed to translate the position of the ACP (Meuleman et al. 2016; van Asseldonk and van der Kooij 2012) to the coordinates of the hip joint, {H}, following which the features of the ACP in {H} were analyzed (Fig. 5). The AJA and ACP of all the subjects were obtained and normalized as a function of the gait cycle percentage, which was 0% corresponding to the initial contact of the concerned leg. The initial-contact and toe-off events were detected by a phase detection method (Zeni et al., 2008). The FPA was calculated as the angle between the foot vector and forward laboratory axis, projected onto the laboratory's transverse plane during the foot flat phase (Simic et al., 2013).

In this study, the coordinates of the pelvis, {P}, were used to establish {H} because the directions of the axes of the two coordinates were the same. The positions of the markers on the pelvis were used to establish {P}. The origin was the middle point from the R.Asis to the L.Asis in the Helen Hayes marker set as shown in Fig. 5. The positive direction of the y axis was directed from the R.Asis to the L.Asis. The z axis was directed inferiorly as the vector that intersects the y axis and perpendicular to the plane containing the L.Asis, R.Asis and sacral markers. The x axis of the pelvis (directed anteriorly) was determined as the cross-product of the y and z axes. Therefore, the description of {P} is:
(1)
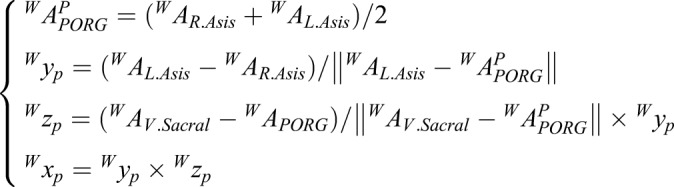
where *^W^A_R.Asis_*, *^W^A_L.Asis_* and *^W^A_V.Scaral_* are the vectors of the R.Asis, L.Asis, and V.Scaral, respectively, *^W^A^P^_PORG_* is the vector of the origin of {*P*}, and *^W^x_p_*, *^W^y_p_* and *^W^z_p_* are the vectors of the base of {P}. They are all described in {*W*}. Thus, the description of {*H*} is:(2)
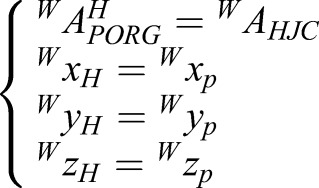
where *^W^A^H^_PORG_* is the vector of the origin of {*H*}. *^W^A_HJC_* is the vector of the HJC and*^W^x_H_*, *^W^y_H_*, and *^W^z_H_* are the vectors of the base of {*H*}. They are all described in {*W*}. {*H*} can be obtained by a homogeneous coordinate transformation, including a rotation and translation of {W}. The homogeneous coordinate transformation can be described as a transformation matrix:(3)
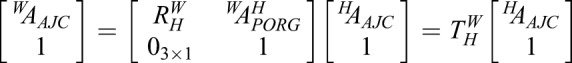
where *T ^W^_H_* is the transformation matrix from {*H*} to {*W*}, *^W^A_AJC_* and *^H^A_AJC_* are the vectors of the ACP in {*W*} and {*H*}, respectively, and *RW H* is the rotation matrix from {*H*} to {*W*}, i.e.:(4)
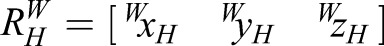
Therefore, *^P^A* is:(5)
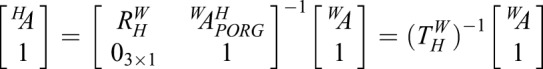


#### Statistical analysis

The gait event and extreme values of the trajectories were extracted as gait features (Figs 3 and 4). Multivariate regression analysis was used to predict gait features. A predicator was adjusted and added to the model based on ANOVA. ANOVA was performed to determine the significant mean differences for comparing the selected gait features in terms of the MP (walking speed) and SPs (body height, foot length, foot width and FPA); subsequently, the predicators for the regression analysis to be performed were chosen after testing the normality and sphericity.

LOOCV was used to test the relationship (Supplementary methods and Fig. S1). In this paper, the maximum number of each variable is taken as three by default. Gait features were analyzed via regression analysis using the equation:(6)
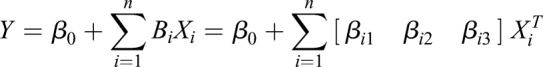
where *Y* represents the relative timing, angle and angular velocity of each gait features. *β*_0_ represents intercept. 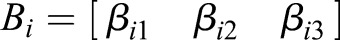 is the coefficient of the predictor *X*_*i*_. *n* indicates the number of the predictors included in the equation. Coding the *B*_*i*_ as *R*_*i*_:(7)
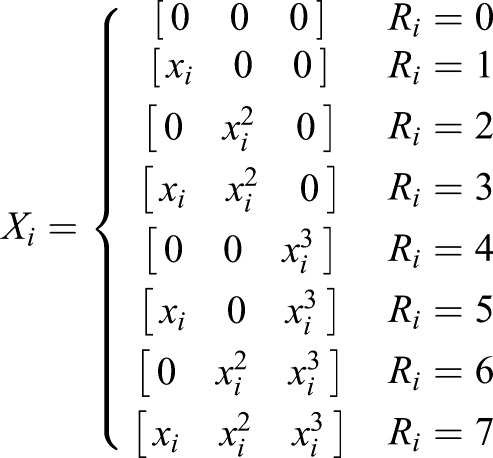
So, the formation of the regression model was:(8)
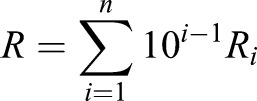
The model with the least MSE at a significance level of *P*=0.05 was chosen. All the statistical analyses were performed using MATLAB R2019 (The MathWorks, Natick, USA).

#### Curve fitting

The obtained regression models were used to reconstruct the reference patterns. First, the values of gait features were calculated. Subsequently, piecewise cubic spline fitting between each pair of consecutive gait features was performed because it creates continuous trajectories in terms of the position and velocity. Each spline is based on four constraints (initial and final position and velocity).

First, the values of the gait features were calculated. Subsequently, piece-wise cubic spline fitting between each pair of consecutive gait features was performed because it creates continuous trajectories in terms of the position and velocity. Each spline is based on four constraints (initial and final position and velocity) and requires a third order polynomial:(9)
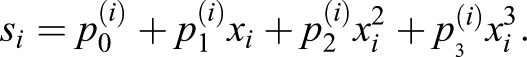
where *s_i_* represent the spline between gait feature *i* and *i*+1 and 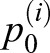 - 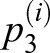 its coefficients. Between (*t*_*i*_, *s*_*i*_) and (*t*_*i*+1_, *s*_*i*+1_), a cubic spline is formulated. The position of the gait features:(10)
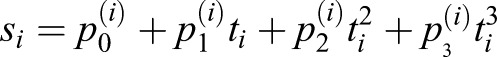
(11)
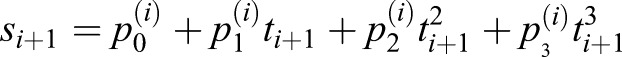
and the velocity of the gait features:(12)
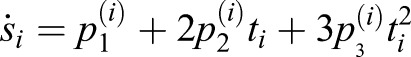
(13)
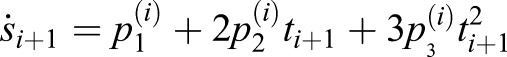
fill in these equations for two subsequent gait features yielding:(14)
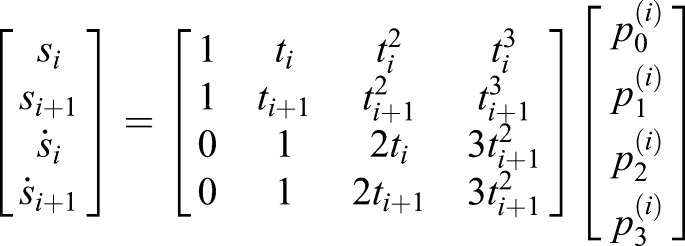
which can be written as:(15)
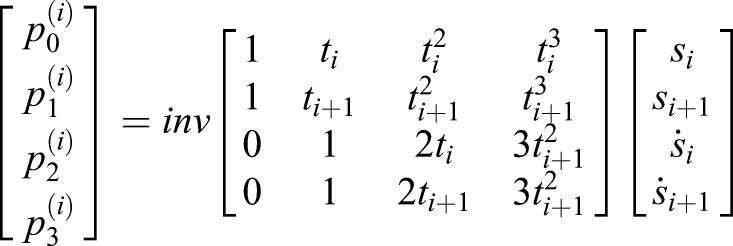


## Supplementary Material

Supplementary information
