# Supplementary material for: Parametric generation of three-dimensional gait for robot-assisted rehabilitation
Source: Biol Open. 2020 Mar 5;9(3):bio047332. doi: 10.1242/bio.047332 (PMC7063668; doi:10.1242/bio.047332)
Supplement: Supplementary information [file biolopen-9-047332-s1.pdf]

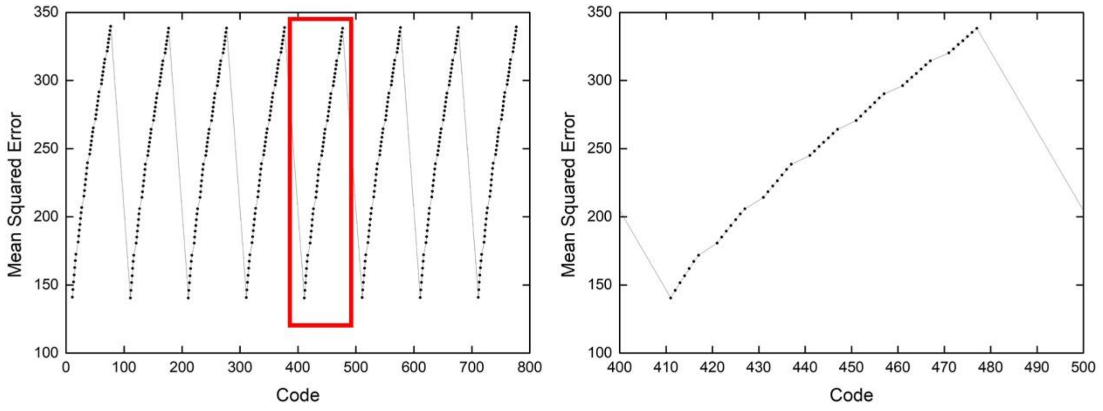

Fig. S1. Selection of the polynomial degree of the predicators via cross-validation. Taking Ye1 and as an example. From the calculation results, it can be seen that the model coding value meeting the conditions was $R = 411$.

Biology Open • Supplementary information

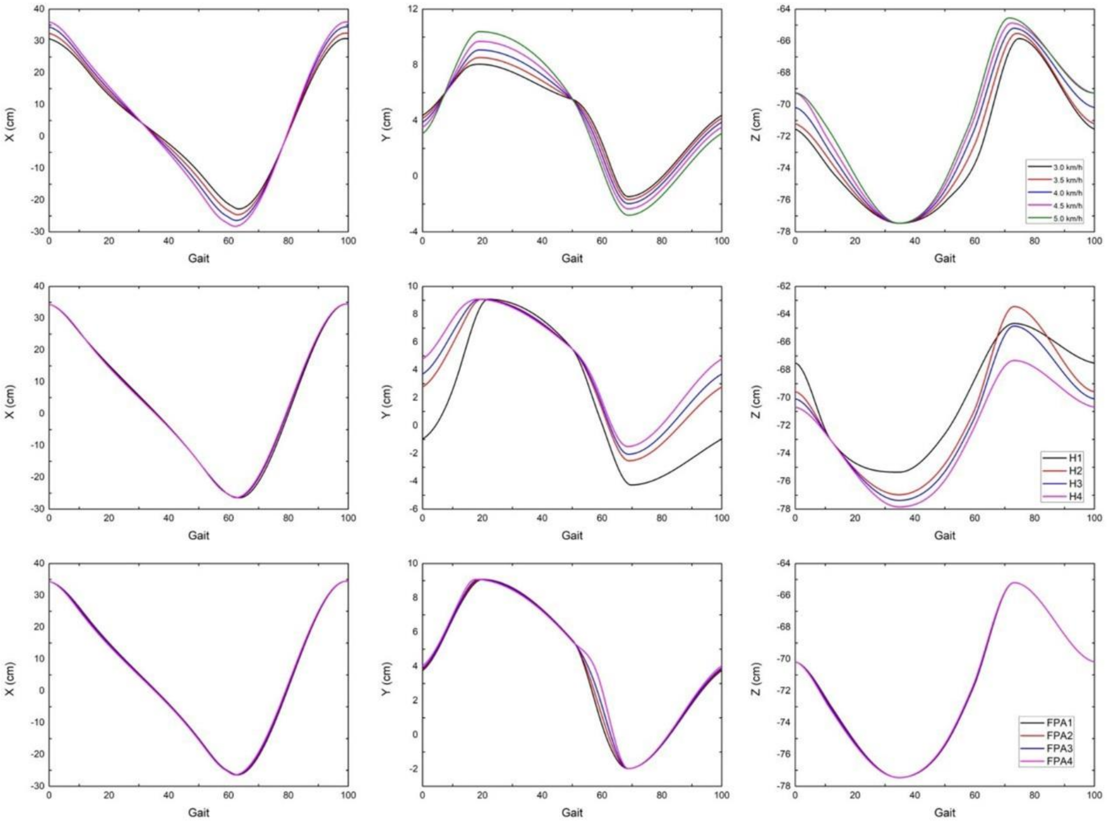

Fig. S2 Generated trajectories based on the FPA, body height, and walking speed

Biology Open • Supplementary information

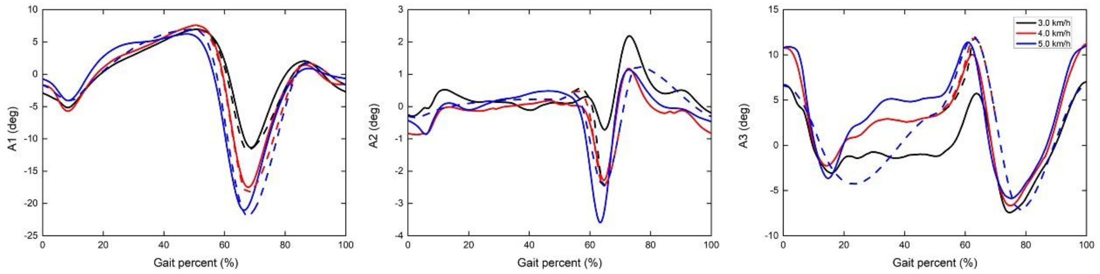

Fig. S3 Generated trajectories versus average trajectories. The generated trajectories (dashed line) are based on the predicted gait features and the average trajectories are averaged across participants (solid line). The trajectories are presented at three different speeds. The generated trajectories are generated for a subject with parameters equal to the mean parameters of all participants (H = 168.750 cm, FPA = 8.320°, L = 24.045 cm, W = 9.680 cm).

Biology Open • Supplementary information

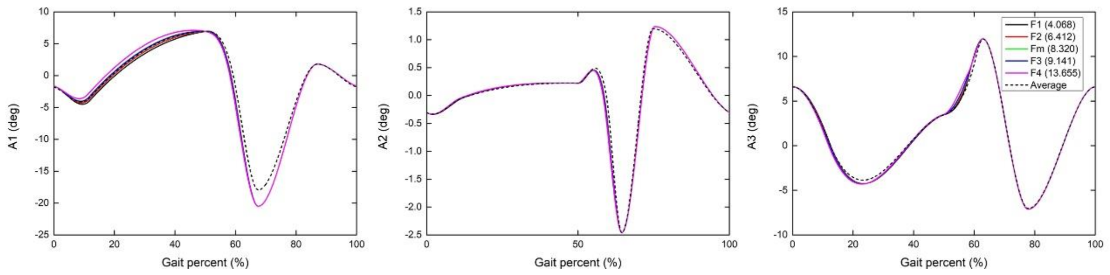

Fig. S4 Generated trajectories versus speed-dependent trajectories. The generated trajectories (solid line) are based on the predicted gait features and the average trajectories are speed-dependent trajectories across participants (dashed line, marked as average). The generated trajectories are presented at five different foot progression angles (F1, F2, F3, F4, and their mean value Fm in degree) with other parameters equal to the mean parameters of all participants (v = 4.0 km/h, H = 168.750cm, L = 24.045 cm, W = 9.680 cm). The speed-dependent trajectories are generated for a subject with parameters equal to the mean parameters of all participants (v = 4.0 km/h, H = 168.750 cm).

Biology Open • Supplementary information

Supplementary Methods

Example: Regression model optimization to predict Ye1
According to the result of ANOVA, both motion (walking speed, $p = 0.002 < 0.005$) and structural parameters (body height, $p = 0.000 < 0.0083$ and FPA, $p = 0.005 < 0.0083$) have significant influence on the gait features, Ye1p and $n = 3$.
Let

$$\begin{cases} x_1 = F \\ x_2 = H \\ x_3 = v \end{cases}$$

The code for single variant:

$$X_1 = \begin{cases} \begin{bmatrix} 0 & 0 & 0 \end{bmatrix} & R_1 = 0 \\ \begin{bmatrix} F & 0 & 0 \end{bmatrix} & R_1 = 1 \\ \begin{bmatrix} 0 & F^2 & 0 \end{bmatrix} & R_1 = 2 \\ \begin{bmatrix} F & F^2 & 0 \end{bmatrix} & R_1 = 3 \\ \begin{bmatrix} 0 & 0 & F^3 \end{bmatrix} & R_1 = 4 \\ \begin{bmatrix} F & 0 & F^3 \end{bmatrix} & R_1 = 5 \\ \begin{bmatrix} 0 & F^2 & F^3 \end{bmatrix} & R_1 = 6 \\ \begin{bmatrix} F & F^2 & F^3 \end{bmatrix} & R_1 = 7 \end{cases}, X_2 = \begin{cases} \begin{bmatrix} 0 & 0 & 0 \end{bmatrix} & R_2 = 0 \\ \begin{bmatrix} H & 0 & 0 \end{bmatrix} & R_2 = 1 \\ \begin{bmatrix} 0 & H^2 & 0 \end{bmatrix} & R_2 = 2 \\ \begin{bmatrix} H & H^2 & 0 \end{bmatrix} & R_2 = 3 \\ \begin{bmatrix} 0 & 0 & H^3 \end{bmatrix} & R_2 = 4 \\ \begin{bmatrix} H & 0 & H^3 \end{bmatrix} & R_2 = 5 \\ \begin{bmatrix} 0 & H^2 & H^3 \end{bmatrix} & R_2 = 6 \\ \begin{bmatrix} H & H^2 & H^3 \end{bmatrix} & R_2 = 7 \end{cases}, X_3 = \begin{cases} \begin{bmatrix} 0 & 0 & 0 \end{bmatrix} & R_3 = 0 \\ \begin{bmatrix} v & 0 & 0 \end{bmatrix} & R_3 = 1 \\ \begin{bmatrix} 0 & v^2 & 0 \end{bmatrix} & R_3 = 2 \\ \begin{bmatrix} v & v^2 & 0 \end{bmatrix} & R_3 = 3 \\ \begin{bmatrix} 0 & 0 & v^3 \end{bmatrix} & R_3 = 4 \\ \begin{bmatrix} v & 0 & v^3 \end{bmatrix} & R_3 = 5 \\ \begin{bmatrix} 0 & v^2 & v^3 \end{bmatrix} & R_3 = 6 \\ \begin{bmatrix} v & v^2 & v^3 \end{bmatrix} & R_3 = 7 \end{cases}$$

The coding is

$$R = R_1 + 10R_2 + 100R_3$$

There are 512 models, each with a unique code.
LOOCV was used to calculate the mean squared error (MSE) of the model as well as the p value. Through calculation, 392 groups met the $p < 0.05$, and the corresponding codes of the group with the smallest root mean square were found. From the calculation results, it can be seen that the model coding value meeting the conditions was $R = 411$ (Fig. S1) and the fitting form of the model was

$$Y = \beta_0 + \beta_{33}x_3^3 + \beta_{21}x_2 + \beta_{12}x_1 = \beta_0 + \beta_{33}v^3 + \beta_{21}H + \beta_{12}F$$

Biology Open • Supplementary information
